# Futility in healthcare among Mexican female patients with breast cancer in advanced stage: The patient perspective

**DOI:** 10.1371/journal.pone.0326332

**Published:** 2025-06-23

**Authors:** María del Mar Yukie Namba-Bando, Irazú Contreras-Yáñez, Juan Alberto Tenorio-Torres, Virginia Pascual-Ramos

**Affiliations:** 1 Fundación de Cáncer de Mama (Breast Cancer Foundation) FUCAM A.C., Mexico City, Mexico; 2 Immunology and Rheumatology Department, Instituto Nacional de Ciencias Médicas y Nutrición Salvador Zubirán, Mexico City, Mexico; 3 Centro Interdisciplinario de Bioética de la Universidad Paramericana (CIBUP), Mexico City, Mexico; Sichuan University West China Medical Center, CHINA

## Abstract

**Background:**

Breast cancer is the leading cause of cancer-related deaths among women in Mexico, and the outcomes for patients are often poor. Futility in healthcare (FHC) occurs when treatment fails to achieve its intended goals, resulting in negative consequences. This study aimed to estimate the proportion of female patients in Mexico with advanced-stage breast cancer (ABC) who perceive FHC. Additionally, we inform the development and validation of a questionnaire (FHC-Q) to assess this phenomenon.

**Patients and methods:**

This cross-sectional study was conducted from May 17, 2024, to December 30, 2024, in three phases. It involved three convenience samples (S) of adult women with ABC: S-1 (n = 30), S-2 (n = 201), and S-3 (n = 257). Phase 1 focused on constructing the FHC-Q, evaluating its content validity (expert agreement), and conducting a pilot test to assess its feasibility, all within S-1. Phase 2 involved assessing the questionnaire’s reliability (internal consistency and temporal stability), construct validity (correlations between FHC-Q and DASS21 scores, along with exploratory factor analysis), and criterion validity (correlations between FHC-Q and SF-36 scores), all in S-2. Phase 3 estimated the FHC phenomenon in S-3. The definition of FHC was established using the Delphi method.

**Results:**

Participants represented typical female Mexican patients with ABC. The FHC-Q comprised 16 items distributed into five dimensions. The FHC-Q demonstrated feasibility and validity, with expert agreement of ≥80% and a five-factor structure explaining 57.8% of the variance. Correlations between the FHC-Q, DASS21 and SF-36 scores were significant and low to moderate. The FHC-Q reliability was confirmed with a Cronbach’s alpha of 0.766 and an ICC of 0.845 (95% CI: 0.735–0.909).

FHC was perceived by 3.9% of the participants

**Conclusions:**

FHC was perceived by a minority of Mexican women with ABC stages. The FHC-Q was valid, reliable and feasible to assess the phenomenon in the target population.

## Introduction

Breast cancer is the leading cause of cancer-related deaths among women globally and in Latin America (LATAM) [1–3]. Factors such as advanced stage at diagnosis [3,4], high tumor grade [4], negative hormone receptors [4], HER-2 amplification [4], tumor subtype [3], and age [3] are linked to higher mortality rates. Additionally, socioeconomic and cultural factors significantly influence survival, affecting access to screening, timely diagnosis, and effective treatments [5–7]. This contributes to the higher mortality of breast cancer in LATAM compared to developed countries [8,9].

The cancer mortality rate in Mexico increased from 58 per 100,000 residents in 1998–67 in 2008 [2]. Since 2006, breast cancer has been the leading cause of cancer deaths among Mexican women, representing 9% of fatalities in 2023 [2,10,11]. By 2030, an estimated 38,420 women will be diagnosed, with 11,781 (30.7%) expected to die from the disease in LATAM, posing a significant challenge for healthcare systems [12]. Factors contributing to poor outcomes include advanced-stage diagnoses [3], a high prevalence of triple-negative breast cancer [3], unique gene expression patterns [3], tumor biology [3], delays in diagnostic procedures [5], and social determinants of health [13,14].

Futility in healthcare (FHC) is a phenomenon recognized across various languages, disciplines, and cultures [15], with roots tracing back to the time of Plato [16]. Although many authors have attempted to define it in clinical contexts, a widely accepted bioethical definition remains elusive [16–21]. The determination of what constitutes meaningful versus futile treatment is inherently subjective, influenced by individual values, beliefs, and experiences [15]. Generally, treatment is deemed futile when it fails to meet its intended goals. However, defining these goals is often challenging within the patient-centered care model, where patients are active participants in their treatment decisions [22,23]. Morata et al. [15] conducted an evolutionary concept analysis to explore the historical development of the FHC concept and its potential future evolution alongside technological and systemic changes in healthcare [24]. They propose a consensus definition for FHC as “complex patient conditions characterized by interventions or procedures that do not achieve meaningful recovery of the primary ailment, based on the patient’s and multidisciplinary team’s healthcare goals, while often maintaining a latent sense of hope.” The consequences of FHC are complex and typically negative, encompassing increased healthcare costs, potential resource rationing, legal challenges, and ethical dilemmas or moral distress [15].

To effectively address the public health challenge of breast cancer, comprehensive research is necessary across clinical, epidemiological, health systems, translational, and bioethical domains [2]. Such research is crucial for identifying and overcoming the specific challenges faced by different countries while also utilizing successful strategies from other regions. The World Health Organization has noted a significant research gap in LATAM countries, primarily due to insufficient funding and support for researchers, emphasizing the need for targeted interventions [25].

Currently, there is no valid, reliable, or objective method to accurately identify or measure FHC from the patient perspective, either quantitatively or qualitatively. This study aims to develop and validate a comprehensive questionnaire to assess FHC (FHC-Q) among female Mexican patients with advanced-stage breast cancer. Additionally, it will estimate the prevalence of this phenomenon in the target population, which represents our primary objective.

## Patients and methods

### Ethical considerations

The study was performed in compliance with the Helsinki Declaration [26]. The Research Ethics Committee of the Fundación para Cáncer de Mama (Breast Cancer Foundation, FUCAM A.C.) approved the study (Reference number: CEI/PI 24–01/2024). All participants provided written informed consent to join the study.

### Study setting, population, and duration

FUCAM A.C. is a non-profit organization in Mexico and LATAM that specializes in breast cancer treatment and monitoring. Since 2005, it has treated over 11,000 women and provided a comprehensive approach that includes early detection, diagnosis, medical and surgical treatment, breast reconstruction, and psychological support [27]. According to its 2022 report, FUCAM conducted 57,089 mammograms, diagnosed 1,297 new breast cancer cases, and performed 72,381 medical consultations [28].

The study was conducted at FUCAM’s high specialty hospital unit in Mexico City from May 17, 2024, to December 30, 2024. Adult women with confirmed advanced-stage breast cancer [29] were invited to participate if they had received treatment at FUCAM or were attending the palliative care unit (inclusion criteria). Advanced-stage breast cancer was defined based on the following three criteria: 1. Clinical Stage IIB with T3 N0 and beyond; 2. Pathologically advanced stage: This refers to cases that may clinically appear to be stage I or II, but reveal more aggressive features upon pathological examination, such as microscopic lymph node involvement or vascular invasion; and 3. Evidence of metastatic disease: This involves cases where the cancer has spread to distant organs, irrespective of the initial clinical or pathological stages.

Patients were approached in waiting areas for consultations related to pain management, psycho-oncology, rehabilitation, nutrition, and various oncology specialties. Those with severe cognitive impairment were excluded.

### Study design

The study utilized a cross-sectional design with three phases and is reported in accordance with the STROBE guidelines (see “Appendix 1. **STROBE checklist**”). Phase 1 involved the construction of the FHC-Q, which included item generation, pilot testing, and feasibility evaluation. Phase 2 assessed the reliability and validity of the FHC-Q. Phase 3 evaluated the prevalence of FHC in the target population according to the FHC-Q (see Appendix 2). We followed established guidelines for developing health measurement scales when no suitable instruments existed [30].

### Description of samples and sample size calculation

This study analyzed three samples (S) of consecutive female outpatients with confirmed advanced-stage breast cancer. During Phases 1 and 2, two samples were used [31,32]: S-1, which included at least 30 participants for pilot testing, and S-2, comprising at least 200 participants for the validation of the FHC-Q. S-3 consisted of 257 participants and was utilized in Phase 3 to meet the primary objective.

We calculated the necessary sample size to detect FHC in the target population based on prevalence rates of 4%, 12%, and 25% for patients meeting three, two, and one criterion of therapeutic aggressiveness, respectively, as reported by Barón-Duarte et al. [33]. Additionally, the International Agency for Research on Cancer reported a 5-year prevalence of breast cancer in Mexico in 2020, with 99,288 cases, of which 66.4% were classified as advanced stages [34]. This results in an estimated 65,927 cases of advanced-stage breast cancer in Mexico. The final required sample size ranged from 59 to 287 patients, with a 95% confidence level and 5% precision, according to the three prevalence rates mentioned. This final sample size allowed us to achieve a confidence level of 95% and a precision of 5% for the primary objective.

Non-probabilistic, intentional sampling was employed, including consecutive women outpatients diagnosed with advanced-stage breast cancer [32].

### Procedures

#### Phase 1- Construction of the conceptual model of the FHC-Q, instructions and items generation, pilot testing, scaling responses, and questionnaire feasibility.

Literature review: A literature review confirmed that there is no valid and reliable tool for assessing FHC [17]. Some prognostic tools have been proposed, such as the Injury Severity Score for trauma patients and the Acute Physiology and Chronic Health Evaluation score for critically ill adults, but these have had limited success [15,35,36]. These tools often lead to false positives and lack the statistical confidence needed for effective care, resulting in their limited adoption [37–39]. Currently, Earle’s aggressiveness criteria [40] are viewed as the gold standard for evaluating therapeutic aggressiveness in oncology for end-of-life care at the institutional level [33]; however, they do not consider the patient perspective. These criteria focus on three areas: the overuse of chemotherapy near death, inappropriate use of devices in the emergency department and ICU, and insufficient or delayed access to hospice and palliative care programs [33]. Accordingly, we decided to develop a new instrument.

Instructions and items generation: Two types of sources were used for instruction and item generation: theoretical literature [15–21] and suggestions from a coauthor with expertise in research, bioethics, and clinical involvement at FUCAM. The initial version of the FHC-Q (v1) was drafted and reviewed by four professionals: a bioethicist, a psycho-oncologist, a surgical oncologist, and a social worker. Their feedback informed the development of a second version (v2), which also included an item for participants to select their preferred term among three options related to the evaluated phenomenon: futility, obstinacy, and lack of therapeutic adequacy.

Version 2 (v2) was tested with eleven patients in four formats, allowing them to choose their preferred presentation style, either as questions or statements with corresponding response scales. The approved format of v2 was then reviewed by an expert committee of 14 members, including surgical oncologists (2), radiation oncologists (2), palliative care specialists (2), medical oncologists (4), a psychologist, a psychiatrist, and bioethicists (2). The committee evaluated the instructions and items based on clarity, appropriateness for the target population, and absence of emotional bias (content validity). Any poorly evaluated instructions or items were revised, leading to the creation of a third version (v3).

Pilot testing: Version 3 (v3) was pilot tested with 30 outpatients from the target population. Participants assessed the clarity of the instructions and items to evaluate face validity, as well as the feasibility of the questionnaire, which included its format, the time required for completion, and the patients’ willingness to fill out the survey. Based on the pilot results, version 4 (v4) was deemed suitable for the validation process (Phase 2).

Scale response: Two types of response scales were provided to match the item formats presented. The response scale could be displayed either as frequency or as a level of agreement, using a 5-point Likert scale in both cases.

### Phase 2. FHC questionnaire validity and reliability

Construct validity was established through convergent validity by examining the correlations between the FHC-Q and the Depression, Anxiety, and Stress Scale-21 (DASS21) scores [41]. Additionally, factor analysis was conducted for further verification [42].

Content validity was evaluated by a committee of experts selected for their experience, reputation, availability, and impartiality [43].

Criterion validity was assessed by correlating FHC-Q scores with those from the 36-item Short Form Survey (SF-36) [44], a validated tool for measuring health-related quality of life (QoL) across diverse populations. The SF-36 encompasses eight scales and two distinct dimensions: the Physical Component Summary (PCS) and the Mental Component Summary (MCS). Scores range from 0 to 100, with higher scores indicating better QoL. Furthermore, MCS and PCS scores were compared between patients who had FHC and those who did not.

Reliability was assessed via internal consistency and temporal stability (test-retest) by administering the questionnaire to 58 patients at baseline and two weeks later [42].

#### Step 3. FHC prevalence.

A modified Delphi exercise [45] was proposed to establish criteria for the prevalence of FHC, based on the results of the FHC-Q. A panel of ten experts was assembled, including two specialists in palliative care, one surgical oncologist, two psycho-oncologists, two bioethicists, and one social worker. The experts participated in three rounds of questions, focusing on ranking the dimensions of the FHC-Q according to their importance for the construct (ordered from first to fifth) and determining their essentiality in addressing the construct (Yes/No).

The FHC-Q was administered alongside standardized assessments to collect sociodemographic (Table 1), disease-related (Table 2), and cancer-related (Table 3) variables. Data were obtained from patients and verified through chart reviews.

**Table 1:** Sociodemographic characteristics of the patients included in the three samples.

	S-1n = 30	S-2n = 201	S-3n = 257
Years of age	50.5 (43-65)	56 (45.5-65.5)	55 (46-63.5)
Years of formal education	12 (9-17)	12 (9-17)	12 (9-17)
Civil status*			
*Married/cohabitant*	14 (46.8)	94 (46.8)	127 (49.5)
*Single*	10 (33.3)	61 (30.3)	75 (29.2)
*Widow*	4 (13.3)	31 (15.4)	33 (12.8)
*Divorced*	2 (6.6)	15 (7.5)	22 (8.5)
Occupation*			
*Formal and non-formal job*	12 (40)	62 (30.8)	90 (35)
*Housewife*	15 (50)	115 (57.2)	140 (54.5)
*Student*	0	3 (1.5)	3 (1.2)
*Other*	3 (10)	21 (10.5)	24 (9.3)
Religious beliefs*	16 (100)¹	165 (82.1)	226 (87.9)
Low socioeconomic status*²	7 (33.3)	56 (27.9)	68 (38.2)

Data presented as median (Q25-Q75) as otherwise indicated. *Number (%) of patients. ¹There were 14 missing data. ²There were 9, 65, and 79 patients, respectively, who did not want to answer. Low socioeconomic status was defined as a family income below $250 per month.

**Table 2:** Disease-related characteristics of the patients included in the three samples.

	S-1 n = 30	S-2 n = 201	S-3 n = 257
**Cancer-related characteristics**			
Months of disease duration	5 (3-17.8)	8 (3-20.5)	11 (4-40)
ECOG performance status scale score	1 (1-2)	1 (1−1)	1 (0-1)
	*MD 7*	*MD 36*	*MD 35*
Patients with ECOG score of 0*	5 (21.7)	40 (24.2)	86 (38.7)
Patients with ECOG score of 1*	14 (60.9)	120 (72.7)	129 (58.1)
Patients with ECOG score of 2*	3 (13)	3 (1.8)	3 (1.4)
Patients with ECOG score of ≥3*	1 (4.3)	2 (1.2)	4 (1.8)
**Comorbidity**			
Patients with ≥ 1 comorbid condition*	14 (46.7)	82 (40.8)	99 (38.5)
Treatment for comorbid conditions*¹	13 (92.9)	72 (87.8)	89 (89.9)
Mental health comorbidity*	2 (6.7)	8 (4)	9 (3.5)
DASS21 score of ≥moderate severity^*,1^	*5 MD*	*15 MD*	*0 MD*
*Depression (≥7)*	6 (24)	41 (22)	61 (23.7)
*Anxiety (≥6)*	8 (32)	59 (31.7)	83 (32.3)
*Stress (≥10)*	4 (16)	28 (15.1)	47 (18.3)
**Patient-reported outcomes measures²**			
HAD-DI score (0–3 scale)	0.5 (0.13-1.13)	0.4 (0-0.9)	0.4 (0-1)
Patients with disability (HAQ-DI score >0.5)*	12 (50)	83 (44.6)	123 (47.9)
SF-36 score			
*PCS*	56.8(50.6-66.6)	59.2 (47.3-76.6)	59.5 (47.4-77.5)
*MCS*	58 (49.6-67.6)	61 (47.9-73.5)	62.5 (48.9-77.6)
Patients with SF-36 score within normal range* (0–100)			
*PCS (≥79)*	2 (8.3)	34 (18.3)	53 (20.6)
*MCS (≥76.7)*	3 (12.5)	42 (22.6)	66 (25.7)
Family APGAR score (0–20 scale)	19 (17-20)	20 (16.5-20)	20 (16-20)
Patients with normal family function*	19 (79.2)	140 (75.3)	192 (74.7)
FHC score (16–80)	68.5 (59-72)	65 (58.5-71)	66 (58-71)
Patients with FHC*	0	9 (4.5)	10 (3.9)
**Treatment-related characteristics**			
Overall treatment intention*			
Palliative	9 (30)	73 (36.3)	72 (28)
Curative	21 (70)	128 (63.7)	185 (72)
Treatment duration, months	4 (2-15.8)	6 (2-17)	9 (2-39)
Time from disease diagnosis and treatment initiation, days	42 (27.8-54.5)	46 (30-59.5)	46 (28-65)
Treatments options* Surgery *Radiation therapy*	15 (50) 9 (30)	99 (49.3) 80 (39.8)	155 (60.3) 132 (51.4)
*Chemotherapy (including targeted therapy and immunotherapy)*	29 (96.7)	191 (95)	242 (94.2)
*Hormone therapy*	8 (26.7)	60 (29.9)	102 (39.7)
*Combined therapy*	17 (56.7)	115 (52.2)	170 (66.1)
Number of treatment options/patient	2 (1-3)	2 (1-3)	3 (1-4)
Chemotherapy type among participants receiving curative chemotherapy*	*N = 19*	*N = 139*	*N = 191*
*Adjuvant*	6 (27.3)	24 (17.3)	39 (20.4)
*Neoadjuvant*	16 (72.7)	110 (79.7)	144 (75.4)
*Combined*	0	5 (3.6)	8 (4.2)
Radiotherapy type among participants receiving curative radiotherapy*	*N = 6*	*N = 60*	*N = 106*
*Adjuvant*	6 (100)	60 (100)	105 (99.1)
*Neoadjuvant*	0	0	0
*Combined*	0	0	1 (0.9)
Number of line of (chemo)therapy/patient among the patients on palliative care	1 (1-4.5)	1 (0.25-2)	1 (0-2)
Number of patients who received re-irradiation among those on palliative care*	1 (3.3)	4 (2)	4 (1.6)

**Table 3:** Breast cancer characteristics.

	S-1 n = 30	S-2 n = 201	S-3 n = 257
Cancer relapse or recurrence* (4, 41 and 39, not applicable; 1, 7 and 8, MD, respectively)	5 (21)	23 (15)	27 (12.9)
Histological type of breast cancer*			
*Invasive lobular carcinoma*	3 (10)	19 (9.5)	22 (8.6)
*Invasive ductal carcinoma*	27 (90)	174 (86.5)	226 (87.9)
*Other types*	0	8 (4)	9 (3.5)
TNM staging system*			
*Stage 0*	0	0	1(0.4)
*Stage I*	0	0	3 (1.2)
*Stage II* *Stage III*	5 (16.7) 20 (66.7)	28 (13.9) 127 (63.2)	40 (15.6) 169 (65.6)
*Stage IV* Non-classifiable	4 (13.3) 1 (3.3)	43 (21.4) 3 (1.5)	41 (16) 3 (1.2)
Molecular subtype*(0, 3 and 3 MD, respectively)			
*Luminal A or HR + /HER2- (HR-positive/HER2-negative)*	11 (36.7)	46 (23.2)	68 (26.7)
*Luminal B or HR + /HER2+ (HR-positive/HER2-positive)*	10 (33.3)	87 (43.9)	112 (44)
*HER2-positive*	1 (3.3)	13 (6.6)	15 (5.9)
*Triple-negative or HR-/HER2- (HR/HER2-negative)*	8 (26.7)	52 (26.3)	59 (23.4)

*Data presented as Number (%) of patients. MD=Missing Data.*

Sociodemographic variables included sex, age, education level, civil status, occupation, religious beliefs, and socioeconomic status. Disease-related characteristics encompassed disease duration, performance status measured by the Eastern Cooperative Oncology Group (ECOG) scale [46], comorbidities, and treatment-related factors. Mental health was assessed using the DASS21 [41], while functional status was evaluated with the Health Assessment Questionnaire Disability Index (HAQ-DI) [47]. Quality of life (QoL) was measured with the SF-36 questionnaire [44], and family functioning was assessed using the Adaptation, Partnership, Growth, Affection, and Resolve index (APGAR) [48].

Cancer treatment-related data included treatment options, the number of treatment modalities per patient, chemotherapy type, lines of therapy (LOT), and instances of reirradiation.

Finally, cancer-related variables included recurrence status, histological type [49], TNM staging [29], and molecular subtype [50].

### Statistical analysis

The FHC score was computed by summing the individual item scores, and the final score ranged from a minimum of 16 to a maximum of 80. This calculation involved recoding items 6, 7, 10, 12, 13, and 15. A lower score reflects a greater patient perception of FHC. The decision to sum item scores aligns with standard practices in questionnaire scoring [51]. Additionally, for the assessment of FHC, we propose scoring the five dimensions of the FHC-Q. It is essential to ensure that at least one item from each dimension has a dichotomous response. This requirement guarantees a thorough evaluation of the FHC construct. By adopting this strategy, we can obtain a numerical representation of the patient’s perceived FHC, facilitating the routine care monitoring of any potential changes over time and the share-decision making process.

Descriptive statistics characterized the patient variables across three samples, with categorical variables reported as frequencies and percentages, and continuous variables summarized using medians and interquartile ranges (Q25-Q75). Statistical comparisons between groups employed the Chi-square (X²) test for categorical variables and the Mann-Whitney U test for continuous variables.

Convergent validity was analyzed using the Spearman rank correlation coefficient (rho) [30]. Construct validity was assessed via exploratory factor analysis (principal components) with Varimax rotation, extracting five factors. Sampling adequacy was confirmed with the Kaiser-Meyer-Olkin (KMO) measure (acceptable value ≥0.5), supported by Bartlett’s test of sphericity (p < 0.05) [52].

Content validity was examined with agreement percentages, and Lawshe/Tristan’s content validity ratio was calculated for individual items and the FHC-Q (mean of individuals’ content validity ratios) [53].

Criterion validity was evaluated using the Spearman rank correlation coefficient (rho) between the FHC-Q and the PCS and MCS scores from the SF-36 [30], as well as by comparing PCS and MCS scores between patients with and without FHC.

Internal consistency of the FHC-Q was assessed using Cronbach’s alpha (α). Temporal stability was determined via intra-class correlation coefficients (ICC) and 95% confidence intervals (CI), utilizing a single measurement, absolute-agreement, 2-way mixed-effects model. Interpretations of Cronbach’s α, ICC, and 95% CI followed established recommendations [54]. Floor and ceiling effects were calculated as the percentage of patients attaining the lowest and highest possible scores.

There were no missing data for the primary outcome; however, the missing data varied from 0% to 23.3% for patient-reported outcome measures (PROMs) in S-1. No imputation was performed.

All statistical analyses were conducted using the Statistical Package for the Social Sciences version 21.0 (SPSS, Chicago, IL), with statistical significance set at p < 0.05.

## Results

### Description of the participants included in the three samples and their breast cancer characteristics

Table 1 summarizes the sociodemographic characteristics of the patients, Table 2 presents the disease-related characteristics and patients’ treatment, and Table 3 details the breast cancer characteristics from the patients included in the three samples. Participants were primarily middle-aged, with a median of 12 years of formal education. Most were housewives, married or cohabiting, with self-reported religious beliefs. A substantial proportion reported a family income of less than $250 per month (equivalent) (Table 1).

Participants in the study had a short duration of illness, with the majority having an ECOG performance status of 1. A significant number of participants also had comorbid conditions, including current mental health disorders of at least moderate severity. While patients’ functional capabilities and overall quality of life were impacted, most reported that their family functioning remained intact. The treatment intention for most patients was curative, and nearly all participants underwent chemotherapy. Details regarding the treatment options for each patient, as well as the types of chemotherapy and radiotherapy used, are summarized in **Table 2**.

Data presented as median (Q25-Q75) as otherwise indicated. *Number (%) of patients. ¹Among those with the condition. ECOG = Eastern Cooperative Oncology Group. HAQ-DI = Health Assessment Questionnaire Disability Index. SF-36 = 36-items short form survey: PCS (Physical Component Summary) and MCS (Mental Component Summary). Family APGAR scale = Adaptation, Partnership, Growth, Affection, and Resolve. FHC = Futility in health care. MD = Missing data. ²There were 7 MD and 17 MD, respectively, but for FHC (no MD).

The predominant histological subtype of breast cancer was invasive ductal carcinoma. Tumors were predominantly classified at stage III according to the TNM staging system and exhibited positive hormone receptor (HR+) status, along with HER2 positivity (**Table 3**).

### Phase 1- Construction of the conceptual model of the FHC-Q, instructions and items generation, pilot testing and scaling responses, and questionnaire feasibility

**Fig 1:**
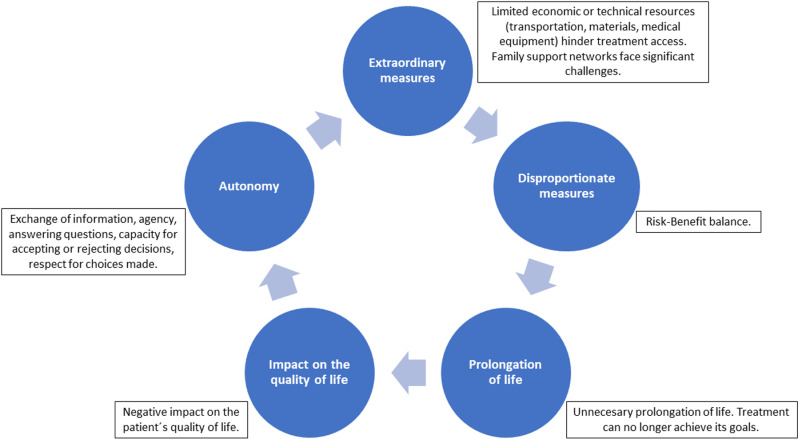
Dimensions and indicators of the theoretical construct of the FHC.

The first version of the FHC questionnaire included 47 items distributed among five dimensions. Fig 1 outlines these five dimensions (extraordinary measures, disproportionate measures, unnecessary prolongation of life, negative impact on the quality of life, and respect for patient autonomy) and their indicators.

The v2 of the FHC questionnaire included 16 items and resulted from a consensus achieved by 100% agreement among the four reviewers involved regarding which items should remain (S1 Table).

The majority of patients (n = 6 [58%]) opted for a version that included statements instead of questions and a frequency scale response based on a 5-point Likert scale. We chose a direct estimation method for the responses, where the scale was defined as follows: 1 = Always, 2 = Almost always, 3 = Sometimes, 4 = Almost never, and 5 = Never.

The results from the multidisciplinary expert committee indicated that there was at least 80% agreement regarding the clarity of the instructions and wording of the items, the appropriateness of the language for the target population, and the absence of emotional bias in the items. However, four members recommended minor changes to item 4 (for better differentiation from item 3) and item 16 (for better differentiation from item 14) to enhance their clarity, and all of these suggestions were adopted (S1 Table). Lawshe/Tristan’s content validity ratio for individual items was ≥ 0.6 and 0.93 for the FHC-Q. The results confirmed content validity.

Results from the pilot testing indicated that overall, patients generally agreed on the clarity of the instructions and items, with over 80% agreement. However, item 2 achieved only 73% agreement regarding its clarity. Some patients found the term “non-financial resources” unclear, although no specific proposals for improvement were suggested. Consequently, the item was updated to include examples.

Most patients (90%) agreed on the FHC-Q format, finding the time required to complete it convenient and expressing a willingness to do so. On average, patients took 18 minutes to complete the questionnaire.

Additionally, 82.4% of the patients and the physicians (14 specialists) directed to provide an answer opted for the term “therapeutic obstinacy” to describe the phenomenon evaluated.

### Phase 2. FHC-Q validity and reliability

#### Construct validity.

Table 4 summarizes Spearman rank correlation coefficients (rho) between the FHC-Q score and specific dimensions scores of the DASS21: depression, anxiety, and stress. Overall, correlations were significant, but their strengths were low.

**Table 4:** Spearman rank correlation coefficients (rho) between the FHC-Q score and specific dimensions scores of the DASS21.

	Depression DASS21	Anxiety DASS21	Stress DASS21
**Rho**	0.350	0.342	0.285
**p-value**	≤0.0001	≤0.0001	≤0.0001

*DASS21 = The Depression, Anxiety and Stress Scale-21 Items*

**Fig 2:**
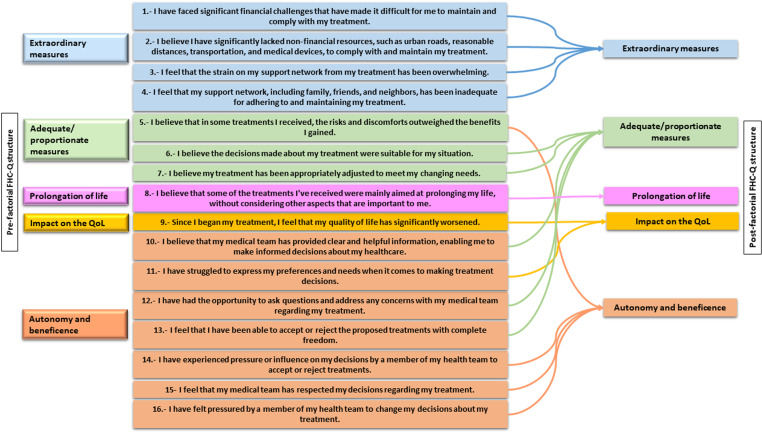
Structure of the FHC-Q previous and post factorial analysis.

The 16 items were distributed into five domains that were renamed as follows: “Extraordinary Measures,” “Adequate/Proportionate Measures” (along with the conditions behind them), “Autonomy and Beneficence,” “Impact on Quality of Life,” and “Prolongation of Life.” The KMO measure was 0.735, and we observed a significant result (X² = 772.549, p ≤ 0.0001) for the Bartlett’s sphericity test. The 5-factor structure accounted for 57.8% of the variance (S2 Table). The structure of the FHC was slightly modified after factorial analysis (Fig 2).

#### Criterion validity.

Table 5 presents the Spearman rank correlation coefficients (rho) between the global FHC-Q score, its dimensions, and the MCS and PCS scores from the SF-36. Overall, the correlations were significant, except for the “Prolongation of Life” dimension of the FHC-Q. Additionally, higher correlations were observed between the FHC-Q and its dimension scores and the MCS of the SF-36 compared to the PCS. The correlation strength was moderate for the “Impact on Quality of Life” dimension of the FHC-Q, while the correlations with the other dimensions were weak. Finally, the MHC and PHC scores were significantly lower in patients with FHC compared to those without: 40.6 (33.3–44) vs. 62.5 (49.6–74.4), p = 0.001 and 45.8 (24.9–48.9) vs. 59.9 (48.3–77.2), p = 0.001, respectively.

**Table 5:** Spearman rank correlation coefficients (rho) between the global FHC-Q score, its dimensions and the MCS and PCS scores of the SF-36.

	MCS	PCS
FHC-Q global score	0.378 (≤0.0001)	0.293 (≤0.0001)
Extraordinary measures	0.231 (0.001)	0.185 (0.008)
Adequate/proportionate measures	0.280 (≤0.0001)	0.189 (0.007)
Autonomy and beneficence	0.217 (0.002)	0.173 (0.014)
Impact on the QoL	0.394 (≤0.0001)	0.384 (≤0.0001)
Prolongation of life	0.111 (0.112)	0.51 (0.473)

*Spearman rank correlation coefficient (and p values) between FHC-Q, its dimensions and the MCS and PCS scores of the SF-36.*

#### Internal consistency and reliability.

Table 6 presents a summary of the internal consistency results (Cronbach’s α, ICC [95% CI]) for the FHC-Q and each of its domains, along with the floor and ceiling effects. The Cronbach’s α value for the FHC-Q was 0.766, while the ICC and its 95% Cl values were 0.845 (0.735–0.909). The mean (±SD) time between the two measurements in the test-retest analysis was 20 days (5.55).

**Table 6:** Psychometric characteristics dimensions that integrated the FHC-Q.

FHC-Q (N° of items; items location)	Cronbach´s α	ICC (95% CI)	Mean of inter-item correlations	Floor/ceiling effects (%)
Extraordinary measures (4; 1–4)	0.697	0.794 (0.649-0.879)	0.365	2.7/3.2
Adequate/proportionate measures (5; 6, 7, 10, 12, 13)	0.693	0.674 (0.444-0.809)	0.311	0.5/43.2
Autonomy and beneficence (4; 5, 14–16)	0.424	0.539 (0.199-0.725)	0.155	0.9/47.1
Impact on the QoL (2; 9, 11)	0.529	0.708 (0.502-0.829)	0.359	3.6/31.1
Prolongation of life (1; 8)	NA	NA	NA	32.9/26.6
FHC-Q (16)	0.766	0.845 (0.735-0.909)	0.170	0.5/0.5

S3 Table indicates that removing individual items did not negatively affect the global Cronbach’s α values for FHC-Q.

### Phase 3. FHC prevalence

#### Delphi exercise results.

Experts reached a consensus on the importance of various factors related to the FHC construct. “Adequate/proportionate measures” ranked first, with 90% agreement among experts, followed by the “(negative) impact on quality of life,” (100% agreement), “(unnecessary) prolongation of life” (70%), “extraordinary measures” (60%), and “autonomy and beneficence” (50% agreement). Additionally, experts identified several key dimensions essential for defining FHC. These include “adequate/proportionate measures” (80% agreement), “(negative) impact on quality of life” (90% agreement), and “(unnecessary) prolongation of life” (80% agreement).

#### FHC definition.

We determined that for a patient to be defined as with FHC, he or she must endorse at least four distinct dimensions from the FHC-Q. This includes all key dimensions previously identified. Furthermore, for each of these four dimensions, the patient must select at least one item response as “Always” or “Almost always.”

#### FHC prevalence.

There were 10 patients (3.9%) who met the FHC definition and this percentage was considered FHC prevalence.

## Discussion

### Considerations on FHC definition

FHC is fundamentally a subjective judgment but remains an essential aspect of everyday clinical practice, with ethical and legal consequences [55]. Achieving a fully objective, concrete, and universal definition of FHC seems unattainable within this framework [56]. Various efforts have been made to define FHC using different approaches, including qualitative and quantitative methods [20,40,57], requiring physicians and patients (or their proxies) to refuse interventions intended to prolong life [56], and more controversial methods based on community [58,59] or institutional [56] standards. However, enforcing an objective definition of FHC could lead to situations where some patients receive interventions or face death due to judgments with which they disagree [56]. In this study, we adopted the approach outlined by Aghabarary et al. [60], which suggests that medical futility should be defined and assessed on an individual level, taking into account the unique circumstances of each case. We integrated this idea into a patient-centered care framework [23], as we endorse that a patient’s perspective and experience cannot be substituted for clinical observations and examinations conducted by specialists [61]. To this end, we developed a questionnaire to evaluate patients’ perceptions of FHC in end-of-life care, complementing existing scientific efforts that capture physicians’ perspectives [62].

### Study strengths

We observed that 3.9% of Mexican women with advanced-stage breast cancer reported experiencing FHC. To address this, we developed and validated the FHC-Q specifically for this population. The creation of the questionnaire followed standardized test-construction methods. The final version demonstrated adequate psychometric properties, including construct, content, and criterion validity, as well as reliability, which was assessed through internal consistency and test-retest evaluations, as recommended [30,52,63]. Patient evaluations indicated that the questionnaire was feasible for use, particularly among individuals with low literacy levels. This suggests that it can be easily applied to patients across the LATAM region and beyond. Patients were also directly involved in establishing the face validity of the questionnaire. While clinicians may have better insights into certain aspects of cancer, such as disease classification, only patients can accurately report on the more subjective elements of their experience [64].

### FHC prevalence

The prevalence rate of FHC in our study is comparable to that reported by Barón-Duarte et al. [33]. In their analysis of therapeutic aggressiveness based on Earle’s criteria, they studied 1,001 patients with advanced cancer who died between 2010 and 2013. The authors found that approximately 25% of the patients met at least one criterion for aggressiveness, while 4% of the patients met all three criteria. However, healthcare specialists have also noted higher rates of FHC in end-of-life care, extending beyond cancer patients [62]. Earle et al. [65] examined Medicare claims for 28,777 patients aged 65 and older who died within a year of a cancer diagnosis between 1993 and 1996. They found that 18.5% were still receiving treatment within two weeks of death, and 9.1% to 9.4% experienced multiple emergency visits or hospitalizations in their final month. In a 1993 study by Solomon et al. [66], 47% of 1,446 healthcare providers across five U.S. hospitals reported acting against their conscience in end-of-life care, with 55% feeling treatments were sometimes overly burdensome. A 2011 survey by Sirovich et al. [67] of 627 U.S. primary care physicians found that 42% believed their patients were overtreated. In a neurological intensive care unit study, Amoroso et al. [68] found that one-third of patients were considered for FHC, increasing to 50% when including loved ones. Chamberlain et al. [69] surveyed 349 physicians from two New York City academic centers, revealing that 91.3% had provided what they considered futile care in the past six months. Variations in prevalence rates among the studies, including ours, may be attributed to differences in how FHC is defined, the years of publication, and the characteristics of the participants. Notably, our study uniquely defines FHC based on patients’ perceptions. Additionally, the patients in our study had a short disease duration, despite being in an advanced stage of the disease. This may significantly shape local patients’ perceptions, particularly because individuals from lower socioeconomic backgrounds frequently encounter obstacles in accessing the Mexican healthcare system and may view any available treatment as a privilege [2,3].

### FHC-Q psychometric properties

The FHC-Q was developed using a rigorous methodological approach. The construct was validated through a literature review [60], a report from the American Medical Association [56], and scientific publications on FHC concept analysis [15,70–73]. Input from healthcare professionals and patients with advanced-stage breast cancer was integral to reframing the construct, ensuring that the questionnaire reflected patient experiences. Content validity was enhanced by involving representative populations during questionnaire development. Item wording underwent optimization through face validity testing with multidisciplinary groups of healthcare experts and patients. The clarity of instructions and the final questionnaire were evaluated by patients in a pilot test before formal validation, leading to essential adjustments that improved patient acceptance and minimized data collection inaccuracies. Three patient samples were utilized, representing typical outpatients from Mexico with advanced-stage breast cancer. The FHC-Q demonstrated adequate internal consistency, with a Cronbach’s α coefficient for the total scale deemed acceptable. Additionally, test-retest reliability assessed in 58 patients by the same researcher showed an ICC with a 95% CI, indicating good reliability [54]. The construct validity was confirmed through KMO sampling and Bartlett’s test of sphericity, both of which validated the sample size’s adequacy for conducting factor analysis [52]. Moreover, the FHC-Q did not show any floor or ceiling effects, defined as occurring when more than 15% of patients score at the lowest or highest possible levels. The presence of floor and ceiling effects reduces the range of data, limiting its variability. This compression can hinder the instrument’s ability to detect differences or changes, as well as impact its reliability and validity. Consequently, it may lead to biased results and incorrect conclusions [30].

### FHC-Q structure

A five-factor model was identified as the most appropriate for the questionnaire, and these five factors together accounted for 57.8% of the total variance. The structure of the FHC-Q underwent modifications from the initial conceptual model. The dimension of “Adequate/Proportionate Measures” was expanded to include three items that were previously categorized under the “Autonomy (and Beneficence)” dimension. Similarly, the “Impact on Quality of Life” dimension was enriched with one additional item. This change may reflect the increasing significance of autonomy as a central principle in the current patient-centered model of care, which permeates other dimensions [74].

The shift towards patient-centered care began with the recognition of patients’ fundamental rights to self-determination and informed consent prior to any intervention. This recognition is now acknowledged in the ethical field as the principle of autonomy. In their book “Principles of Biomedical Ethics,” Beauchamp and Childress [75] establish a framework for medical practice that articulates both utilitarian and deontological principles. They propose a non-hierarchical approach based on four principles: autonomy, beneficence, non-maleficence, and justice, all of which are viewed as absolute obligations. This framework has significantly influenced ethical standards in the US and LATAM [76]. However, Western societies have often overemphasized the principle of autonomy over the other principles [77,78], contributing to the ethical lattice upon which physicians base their decisions.

### Rational for the questionnaire scoring system and the FHC prevalence

Finally, we propose a scoring system for the questionnaire that includes a total score and specific criteria to establish perceived FHC, ensuring its applicability in routine clinical practice. To develop this system, we utilized the Delphi technique, which has proven effective in addressing various critical issues across different health professions. The combination of anonymity, iterative feedback, controlled responses, and the statistical aggregation of group opinions makes this method particularly suitable for tackling emerging and less understood topics, as well as predicting important issues in medicine [79]. Moreover, as a consensus technique based on expert opinions, the Delphi method has the potential to lead to one of the first peer-reviewed publications in this new field, ultimately influencing a substantial body of literature [79].

### Study limitations

Several limitations need to be addressed. First, the FHC-Q was defined solely from the patient’s perspective, without considering the opinions of physicians. It has been suggested that FHC should be approached as a shared decision-making process [33], and the provision of futile care should not only be viewed as an individual patient decision but also in a broader context [80]. Furthermore, while the proposed scoring system is easy to use in a busy clinical setting, it could potentially influence study results [81]. Second, the study focused on Spanish-speaking patients from a single center in Mexico City, which may limit the generalizability of the results to other Mexican or Latin American patients. Future research could benefit from including multicenter recruitment. Third, while the patients in this study were representative of typical Mexican women with breast cancer, the research lacked representation across the entire spectrum of patients with advanced stages of the disease. Fourth, the cross-sectional nature of our study restricts our ability to draw causal inferences. Finally, we assessed only a limited number of psychometric characteristics of the questionnaire, which were considered necessary for an initial evaluation; however, additional relevant factors, such as sensitivity to change, should also be defined in future studies.

### Study implications

The current study investigates the prevalence of FHC among women with advanced-stage breast cancer and introduces a questionnaire to assess this construct.

Breast cancer is the leading cause of cancer deaths among Mexican women, who frequently present with advanced stages at their first consultation, highlighting the study’s public health relevance.

The FHC-Q demonstrated adequate psychometric properties and can be easily scored manually, allowing for immediate patient feedback. This enhances patient-centered care, which the Institute of Medicine defines as care that is respectful of and responsive to individual patient preferences, guiding clinical decisions [23]. This approach has been shown to improve treatment safety, effectiveness, adherence, and health-related outcomes [82], underlining the clinical implications of the study.

Moreover, the patient-centered care model emphasizes respecting patients as unique individuals, advocating for their autonomy and the consideration of their values [82], which further supports the study’s ethical implications.

## Conclusions

The FHC was recognized by a small group of Mexican women who were suffering from advanced stages of breast cancer. The FHC-Q demonstrated strong psychometric properties for assessing this issue within the targeted population. In the context of patient-centered care, it is important that FHC encompasses more than just the physician’s perspective. The patient’s views must also be acknowledged and integrated to fully facilitate shared decision-making.

## Supporting information

SI Appendix STROBE checklist. (PDF)

S2 Appendix Futility in healthcare Questionnaire (FHC-Q). (PDF)

S1 Table Successive FHC questionnaire versions. (DOCX)

S2 Table FHC-Q factorial Matrix. (DOCX)

S3 Table Cronbach alfa values of the FHC-Q when items are removed. (DOCX)
