# Supplementary material for: Futility in healthcare among Mexican female patients with breast cancer in advanced stage: The patient perspective
Source: PLoS One. 2025 Jun 23;20(6):e0326332. doi: 10.1371/journal.pone.0326332 (PMC12185015; doi:10.1371/journal.pone.0326332)
Supplement: S2 Appendix — (PDF) [file pone.0326332.s002.pdf]

Supplementary material Appendix 2. Futility in healthcare Questionnaire (FHC-Q)

Original version

PERCEPCIÓN SOBRE LA OBSTINACIÓN TERAPÉUTICA EN EL TRATAMIENTO DE CÁNCER DE MAMA

Estimado(a) paciente, le pedimos que conteste el siguiente cuestionario que evalúa la Obstinación Terapéutica en pacientes con cáncer de mama.

La Obstinación Terapéutica (o falta de adecuación del esfuerzo terapéutico) se refiere a la aplicación de tratamientos extraordinarios (intensivos) y desproporcionados que prolongan la vida, generan sufrimiento innecesario y afectan negativamente la calidad de vida de los pacientes. Es por ello que para evitarla se busca adecuar los tratamientos en cada paciente, pudiendo tener que retirar o no iniciar medidas terapéuticas consideradas como inútiles en situaciones específicas.

Este cuestionario investiga si usted ha sentido obstinación terapéutica durante su tratamiento para el cáncer de mama.

INSTRUCCIONES:
Para cada frase, seleccione marcando con una cruz dentro del recuadro una sola respuesta, con respecto a que tantas veces está o ha estado de acuerdo.

1. He tenido **dificultades económicas muy importantes** para cumplir y mantener mi tratamiento

☐ Siempre
☐ Casi siempre
☐ Algunas veces
☐ Casi nunca
☐ Nunca

2. Siento que me **han faltado de forma muy importante recursos NO económicos** (como vías urbanas, carreteras o distancia razonables, transporte, aparatos o instrumental médico, etc.) para cumplir y mantener mi tratamiento.

☐ Siempre
☐ Casi siempre
☐ Algunas veces
☐ Casi nunca
☐ Nunca

3. Considero que la **carga** para mis familiares, amigos y vecinos por mi tratamiento ha sido **mucho mayor a nuestras posibilidades.**

☐ Siempre
☐ Casi siempre
☐ Algunas veces
☐ Casi nunca
☐ Nunca

4. Considero que la **ayuda** de mis familiares, amigos y vecinos ha sido **insuficiente** para cumplir y mantener mi tratamiento.

☐ Siempre
☐ Casi siempre
☐ Algunas veces
☐ Casi nunca
☐ Nunca

5. Considero que en alguno de los tratamientos que recibí **los riesgos o las molestias fueron mayores a los beneficios** que obtuve.

- ☐ Siempre
- ☐ Casi siempre
- ☐ Algunas veces
- ☐ Casi nunca
- ☐ Nunca

6. Siento que las **decisiones** que se han tomado frente a mi tratamiento han sido **adecuadas a la situación** en la que me encontraba.

- ☐ Siempre
- ☐ Casi siempre
- ☐ Algunas veces
- ☐ Casi nunca
- ☐ Nunca

7. Considero que mis tratamientos se han **ajustado** de forma adecuada **a medida que ha cambiado mi situación y necesidades.**

- ☐ Siempre
- ☐ Casi siempre
- ☐ Algunas veces
- ☐ Casi nunca
- ☐ Nunca

8. Siento que algunos de los tratamientos que he recibido estaban destinados principalmente a **prolongar mi vida sin tener en cuenta otros aspectos importantes para mí.**

- ☐ Siempre
- ☐ Casi siempre
- ☐ Algunas veces
- ☐ Casi nunca
- ☐ Nunca

9. Siento que mi **calidad de vida ha empeorado considerablemente** desde que comencé mi tratamiento.

- ☐ Siempre
- ☐ Casi siempre
- ☐ Algunas veces
- ☐ Casi nunca
- ☐ Nunca

10. Creo que la **información proporcionada por mi equipo médico ha sido comprensible y útil** para tomar decisiones sobre mi atención médica.

- ☐ Siempre
- ☐ Casi siempre
- ☐ Algunas veces
- ☐ Casi nunca
- ☐ Nunca

11. He experimentado **dificultades para expresar mis preferencias y necesidades** en relación con la toma de decisiones de mi tratamiento.

- ☐ Siempre
- ☐ Casi siempre
- ☐ Algunas veces
- ☐ Casi nunca
- ☐ Nunca

12. Se me ha dado la **oportunidad de hacer preguntas y aclarar dudas** con mi equipo médico en relación con mi tratamiento.

- ☐ Siempre
- ☐ Casi siempre
- ☐ Algunas veces
- ☐ Casi nunca
- ☐ Nunca

13. Siento que **he podido aceptar o rechazar** los tratamientos propuestos **con total libertad**.

- ☐ Siempre
- ☐ Casi siempre
- ☐ Algunas veces
- ☐ Casi nunca
- ☐ Nunca

14. He experimentado **presiones** o influencias en mis decisiones por algún miembro de mi equipo de salud para **aceptar o rechazar** tratamientos

- ☐ Siempre
- ☐ Casi siempre
- ☐ Algunas veces
- ☐ Casi nunca
- ☐ Nunca

15. Siento que mi equipo médico **ha respetado mis decisiones** en relación con mi tratamiento.

- ☐ Siempre
- ☐ Casi siempre
- ☐ Algunas veces
- ☐ Casi nunca
- ☐ Nunca

16. Me he sentido presionada por algún miembro de mi equipo de salud para **CAMBIAR** las decisiones que ya había tomado, sobre mi tratamiento.

- ☐ Siempre
- ☐ Casi siempre
- ☐ Algunas veces
- ☐ Casi nunca
- ☐ Nunca

__English translation__

PERCEPTION OF THERAPEUTIC OBSTINANCY IN BREAST CANCER TREATMENT

Dear patient, we kindly ask you to complete the following questionnaire, which evaluates the concept of Therapeutic Obstinacy in breast cancer patients.

Therapeutic Obstinacy (or inadequacy of the therapeutic effort) refers to the application of extraordinary (intensive) and disproportionate treatments that prolong life but may cause unnecessary suffering and negatively impact the quality of life for patients. <u>For this reason, to avoid it, the aim is to adapt the treatments to each patient, and it may be necessary to withdraw or not initiate therapeutic measures considered useless in specific situations.</u>

This questionnaire investigates whether you have felt therapeutic obstinacy during your treatment for breast cancer.

INSTRUCTIONS: For each statement, select only one answer by marking a cross inside the box regarding how many times you agree or have agreed.

1. I have faced **significant financial challenges** that have made it difficult for me to maintain and comply with my treatment.

☐ Always
☐ Almost always
☐ Sometimes
☐ Almost never
☐ Never

2. I believe **I have significantly lacked non-financial resources**, such as urban roads, reasonable distances, transportation, and medical devices, to comply with and maintain my treatment.

☐ Always
☐ Almost always
☐ Sometimes
☐ Almost never
☐ Never

3. I feel that the **strain** on my support network from my treatment **has been overwhelming**.

☐ Always
☐ Almost always
☐ Sometimes
☐ Almost never
☐ Never

4. I feel that **my support network**, including family, friends, and neighbors, **has been inadequate** for adhering to and maintaining my treatment.

☐ Always
☐ Almost always
☐ Sometimes
☐ Almost never
☐ Never

5. I believe that in some treatments I received, the **risks and discomforts outweighed the benefits I gained**.

☐ Always
☐ Almost always
☐ Sometimes
☐ Almost never
☐ Never

6. I believe **the decisions** made about my treatment **were suitable** for my situation.

☐ Always
☐ Almost always
☐ Sometimes
☐ Almost never
☐ Never

7. I believe **my treatment has been appropriately adjusted** to meet my changing needs.

☐ Always
☐ Almost always
☐ Sometimes
☐ Almost never
☐ Never

8. I believe that some of the treatments I've received were mainly aimed at **prolonging my life, without considering other aspects** that are **important to me**.

☐ Always
☐ Almost always
☐ Sometimes
☐ Almost never
☐ Never

9. Since I began my treatment, I feel that **my quality of life has significantly worsened**

☐ Always
☐ Almost always
☐ Sometimes
☐ Almost never
☐ Never

10. I believe that my medical team **has provided clear and helpful information**, enabling me to make informed decisions about my health care.

☐ Always
☐ Almost always
☐ Sometimes
☐ Almost never
☐ Never

11. I have **struggled to express my preferences and needs** when it comes to making treatment decisions.

- ☐ Always
- ☐ Almost always
- ☐ Sometimes
- ☐ Almost never
- ☐ Never

12. I have had **the opportunity to ask questions and address any concerns** with my medical team regarding my treatment.

- ☐ Always
- ☐ Almost always
- ☐ Sometimes
- ☐ Almost never
- ☐ Never

13. I feel that I have been able to **accept or reject the proposed** treatments **with** complete **freedom**.

- ☐ Always
- ☐ Almost always
- ☐ Sometimes
- ☐ Almost never
- ☐ Never

14. I have experienced **pressure or influence** on my decisions by a member of my health team **to accept or reject** treatments.

- ☐ Always
- ☐ Almost always
- ☐ Sometimes
- ☐ Almost never
- ☐ Never

15. I feel that my medical team has **respected my decisions** regarding my treatment.

- ☐ Always
- ☐ Almost always
- ☐ Sometimes
- ☐ Almost never
- ☐ Never

16. I have felt **pressured** by a member of my health team **to CHANGE my decisions** about my treatment.

- ☐ Always
- ☐ Almost always
- ☐ Sometimes
- ☐ Almost never
- ☐ Never
