# Supplementary material for: Futility in healthcare among Mexican female patients with breast cancer in advanced stage: The patient perspective
Source: PLoS One. 2025 Jun 23;20(6):e0326332. doi: 10.1371/journal.pone.0326332 (PMC12185015; doi:10.1371/journal.pone.0326332)
Supplement: S2 Table — (DOCX) [file pone.0326332.s004.docx]

**Supplementary Table 2.** **FHC-Q factorial Matrix.**

|  | **F-1** | **F-2** | **F-3** | **F-4** | **F-5** |
| --- | --- | --- | --- | --- | --- |
| 1.- I have faced significant financial challenges that have made it difficult for me to maintain and comply with my treatment. | 0.816 |  |  |  |  |
| 2.- I believe I have significantly lacked non-financial resources, such as urban roads, reasonable distances, transportation, and medical devices, to comply with and maintain my treatment. | 0.519 |  |  |  |  |
| 3.- I feel that the strain on my support network from my treatment has been overwhelming. | 0.833 |  |  |  |  |
| 4.- I feel that my support network, including family, friends, and neighbors, has been inadequate for adhering to and maintaining my treatment. | 0.654 |  |  |  |  |
| 5.- I believe that in some treatments I received, the risks and discomforts outweighed the benefits I gained. |  |  | 0.464 |  |  |
| 6.- I believe the decisions made about my treatment were suitable for my situation. |  | 0.758 |  |  |  |
| 7.- I believe my treatment has been appropriately adjusted to meet my changing needs. |  | 0.716 |  |  |  |
| 8.- I believe that some of the treatments I've received were mainly aimed at prolonging my life, without considering other aspects that are important to me. |  |  |  |  | 0.706 |
| 9.- Since I began my treatment, I feel that my quality of life has significantly worsened. |  |  |  | 0.808 |  |
| 10.- I believe that my medical team has provided clear and helpful information, enabling me to make informed decisions about my healthcare. |  | 0.759 |  |  |  |
| 11.- I have struggled to express my preferences and needs when it comes to making treatment decisions. |  |  |  | 0.567 |  |
| 12.- I have had the opportunity to ask questions and address any concerns with my medical team regarding my treatment. |  | 0.530 |  |  |  |
| 13.- I feel that I have been able to accept or reject the proposed treatments with complete freedom. |  | 0.460 |  |  |  |
| 14.- I have experienced pressure or influence on my decisions by a member of my health team to accept or reject treatments. |  |  | 0.595 |  |  |
| 15- I feel that my medical team has respected my decisions regarding my treatment. |  |  | 0.608 |  |  |
| 16.- I have felt pressured by a member of my health team to change my decisions about my treatment. |  |  | 0.859 |  |  |

**F=Factor**
