# Supplementary material for: Futility in healthcare among Mexican female patients with breast cancer in advanced stage: The patient perspective
Source: PLoS One. 2025 Jun 23;20(6):e0326332. doi: 10.1371/journal.pone.0326332 (PMC12185015; doi:10.1371/journal.pone.0326332)
Supplement: S3 Table — (DOCX) [file pone.0326332.s005.docx]

**Supplementary Table 3.** **Cronbach alfa values of the FHC-Q when items are removed.**

| **Items of FHC-Q** | **Cronbach α**  **value** |
| --- | --- |
| 1.- I have faced significant financial challenges that have made it difficult for me to maintain and comply with my treatment. | 0.740 |
| 2.- I believe I have significantly lacked non-financial resources, such as urban roads, reasonable distances, transportation, and medical devices, to comply with and maintain my treatment. | 0.740 |
| 3.- I feel that the strain on my support network from my treatment has been overwhelming. | 0.727 |
| 4.- I feel that my support network, including family, friends, and neighbors, has been inadequate for adhering to and maintaining my treatment. | 0.736 |
| 5.- I believe that in some treatments I received, the risks and discomforts outweighed the benefits I gained. | 0.734 |
| 6.- I believe the decisions made about my treatment were suitable for my situation. | 0.748 |
| 7.- I believe my treatment has been appropriately adjusted to meet my changing needs. | 0.751 |
| 8.- I believe that some of the treatments I've received were mainly aimed at prolonging my life, without considering other aspects that are important to me. | 0.760 |
| 9.- Since I began my treatment, I feel that my quality of life has significantly worsened. | 0.743 |
| 10.- I believe that my medical team has provided clear and helpful information, enabling me to make informed decisions about my healthcare. | 0.749 |
| 11.- I have struggled to express my preferences and needs when it comes to making treatment decisions. | 0.726 |
| 12.- I have had the opportunity to ask questions and address any concerns with my medical team regarding my treatment. | 0.748 |
| 13.- I feel that I have been able to accept or reject the proposed treatments with complete freedom. | 0.736 |
| 14.- I have experienced pressure or influence on my decisions by a member of my health team to accept or reject treatments. | 0.747 |
| 15- I feel that my medical team has respected my decisions regarding my treatment. | 0.746 |
| 16.- I have felt pressured by a member of my health team to change my decisions about my treatment. | 0.744 |
